# Supplementary material for: Systematic review and meta-analysis of late auditory evoked potentials as a candidate biomarker in the assessment of tinnitus
Source: PLoS One. 2020 Dec 17;15(12):e0243785. doi: 10.1371/journal.pone.0243785 (PMC7746183; doi:10.1371/journal.pone.0243785)
Supplement: S1 Appendix — (DOCX) [file pone.0243785.s001.docx]

**Appendix S1**

1. Exclusion of papers for meta-analysis

Of the eleven studies excluded for quantitative analysis, four reported longitudinal differences in LAEP components in randomized trials of tinnitus treatments (1-4). Due to the relatively low number of longitudinal studies in the identified records and the inconsistent use of different LAEP components in these four studies, these papers were excluded for the meta-analysis. Similarly, papers only reporting computed LAEP components such as the mismatch negativity (MMN) (5-7) or contingent negative variation (CNV) (8) were excluded from the statistical analysis due to considerable heterogeneity of the used experimental paradigms. Furthermore, one paper could not be included in the meta-analysis because no measure of variation on the results was reported (9), whereas the study of Ku et al. was excluded due to the use of a highly deviant experimental paradigm (10). Lastly, one paper of the Campbell group (11) was excluded as the authors reported the use of the same dataset in a more recent paper (12). Although these papers were not considered for the final multivariate meta-analysis, they were included in a narrative review.

An overview of the reported outcomes of these studies is presented in S1 Table. Regarding the MMN, no consistent differences between tinnitus patients and controls were identified across studies, as both increased and decreased MMN amplitudes were reported by different authors (6, 13). As only two groups assessed the contingent negative variation (CNV), no meaningful conclusions could be drawn regarding this component. The two studies excluded for meta-analysis due to incomplete reporting or deviating protocol did not report any data contradicting findings of the multivariate model (9, 10).

**S1 Table. Overview of all studies excluded for the quantitative meta-analysis.**

| *First author (year)* | *LAEP component* | *Reason for exclusion* | *Main results* |
| --- | --- | --- | --- |
| **Weisz (2004)** | Mismatch negativity (MMN) | Too much variability in MMN paradigms | Lesion-edge frequency deviants, but not other frequency deviants, elicited abnormal responses from tinnitus patients compared to controls |
| **Kropp (2012)** | Contingent negative variation (CNV) | Not enough studies to compare results with | CNV amplitudes were increased in tinnitus patients compared to controls |
| **Mahmoudian (2013)** | Mismatch negativity (MMN) | Too much variability in MMN paradigms | MMN amplitude for frequency, duration and silent gap deviants was smaller in tinnitus patients than controls |
| **Mahmoudian (2015)** | Mismatch negativity (MMN) | No comparison tinnitus – control (longitudinal study) | MMN amplitude and area under the curve for frequency, duration and intensity deviants were bigger after auditory electrical stimulation |
| **Li (2016)** | Mismatch negativity (MMN) | Too much variability in MMN paradigms; no measures of variation on peak values reported | A subtle frequency deviant elicited a larger MMN in tinnitus patients compared to the control group |
| **Tugumia (2016)** | P300 | No comparison tinnitus – control (longitudinal study) | P300 latency was decreased after an auditory training program, but the decrease was not statistically significant |
| **Ku (2017)** | N100-P200 amplitude | Deviating experimental paradigm: gap detection of gaps with varying lengths | No significant differences between tinnitus patients and control subjects were found |
| **Wise (2017)** | N100 and P200 | No comparison tinnitus – control (longitudinal study) | N100 latency was significanty decreased after a 20-day perceptual training game |
| **Campbell (2018)** | P50 and N100 | Dataset was reused in a more recent paper by the same authors (12) | A lack of gating was observed in the tinnitus group compared to control subjects |
| **El-Minawi (2018)** | Mismatch negativity (MMN) | Too much variability in MMN paradigms | MMN amplitude for frequency deviants was larger in tinnitus patients than controls and latency was shorter. After TRT, tinnitus subjects’ MMN amplitudes and latencies were no longer significantly different from controls |
| **Jacquemin (2019)** | N100, P200, N200 and P300 | No comparison tinnitus – control (longitudinal study) | N100, P200 and N200 latencies were significantly decreased and N200 amplitude significantly increased after 8 biweekly sessions of high-definition trancranical direct current stimulation (HD-tDCS) |

1. Demographic details of participants in included studies

An overview of the gender distribution, mean ages and hearing levels of participants in the included studies is provided in S2 Table.

**S2 Table: Overview of demographic variables of participants in all studies included for the final meta-analysis.**

| *First author  (year)* | | *Number of  participants* | | *Proportion of men* | | *Age  (mean [range])* | | *Hearing levels  (PTA [SD])* | |
| --- | --- | --- | --- | --- | --- | --- | --- | --- | --- |
|  |  | *Tin* | *Con* | *Tin* | *Con* | *Tinnitus* | *Control* | *Tinnitus* | *Control* |
| Shiraishi (1991) (14) | | 20 | 20 | 0.75 | 0.85 | 41.4 [21-60] | 37 [24-60] | 11.93 [12.95] |  |
| Attias (1993) (15) | | 12 | 12 | 1 |  | [26-45] |  | 15.3 [17.58] | 17.82 [11.94] |
| Attias (1996) (16) | | 21 | 21 | 1 |  | 35 [21-45] |  | 24.17 [16.97] | 23.77 [18.67] |
| Jacobson (1996) (17) | | 37 | 15 | 0.76 | 0.6 | 48 | 27.4 |  |  |
| Norena (1999) (18) | |  | 13 |  | 0.38 |  | 53 [20-66] |  |  |
|  | Bilateral tinnitus | 16 |  | 0.56 |  | 52 [30-72] |  |  |  |
|  | Unilateral tinnitus | 9 |  | 0.78 |  | 51 [31-71] |  |  |  |
| Jacobson (2003) (19) | | 32 | 31 | 0.59 | 0.39 | 46 | 39 | 12.83 [10.92] |  |
| Walpurger (2003) (20) | | 10 | 10 | 0.6 | 0.5 | 32.7 [21-44] | 31.1 [21-42] |  |  |
| Dornhoffer (2006) (21) | | 29 | 35 | 0.48 | 0.4 | 54.8 [34-78] | 49.5 [30-80] |  |  |
| Delb (2008) (22) | |  | 10 | 0.70 | 0.6 | 48.26 | [22-29] |  |  |
|  | High distress | 15 |  |  |  |  |  | 21.7 [9.25] |  |
|  | Low distress | 26 |  |  |  |  |  | 18.5 [10.89] |  |
| Santos Filha (2010) (23) | | 30 | 30 | 0.87 | 0.87 | 41 [27-50] | 41.6 [27-50] |  |  |
| Gabr (2011) (24) | | 40 | 40 | 0.48 | 0.5 | 37.3 | 38.5 |  |  |
| Yang (2013) (25) | | 20 | 16 | 0.5 | 0.38 | 43.2 | 42.5 |  |  |
| Houdayer (2015) (26) | | 17 | 17 | 0.65 | 0.47 | 43.4 | 45.7 |  |  |
| Hong (2016) (27) | | 15 | 15 | 0.53 | 0.53 | 30.2 [17-41] | 28.7 [20-43] |  |  |
| Gopal (2017) (28) | | 10 | 10 | 0.5 | 0.5 | 48.9 | 49.8 | 20.8 [16.96] | 17.08 [19.38] |
| Mannarelli (2017) (29) | | 20 | 20 | 0.4 | 0.4 | 50.1 | 49.4 |  |  |
| Asadpour (2018) (30) | | 15 | 6 | 0.67 | 0.5 | 39 | 27 |  |  |
| Durai (2018) (31) | | 16 | 14 | 0.38 | 0.43 | 53.44 [32-76] | 50.25 [22-78] | 28.92 [8.01] | 28.17 [5.98] |
| Morse (2018) (32) | | 13 | 13 | 0.54 | 0.54 |  |  | 22.97 [18.61] | 23.83 [18.72] |
| Campbell (2019) (12) | | 21 | 45 | 0.33 | 0.29 | 23.43 [17-43] | 21.51 [18-33] | 5.33 [6.62] | 5.03 [5.24] |
| Majhi (2019) (33) | | 55 | 51 | 0.45 | 0.45 | 42.91 | 41.63 |  |  |

Tin: Tinnitus group; Con: Control group.

1. Investigation of influential studies

Influential studies or outliers for each LAEP component were identified based on Cook’s distances. An overview of the identified influential studies is provided in S3 Table.

**S3 Table: Influential studies identified for each LAEP component.**

| **Component** | **Influential studies** | **Results after removal of influential studies** |
| --- | --- | --- |
| P50 amplitude | None | N/A |
| N100 amplitude | Jacobson et al., 1996; Delb et al., 2008 | ‘Age difference’ is no longer a significant moderator; no significant difference in N100 amplitude between tinnitus patients and controls |
| N100 latency | None | N/A |
| P200 amplitude | Walpurger et al., 2003; Houdayer et al., 2015 | No differences compared to the primary analysis |
| P200 latency | Walpurger et al., 2003 | No differences compared to the primary analysis |
| N100-P200 amplitude | None | N/A |
| P300 amplitude | None | N/A |
| P300 latency | Majhi et al., 2019 | No differences compared to the primary analysis |

In no case did removal of influential papers result in a different outcome for these post hoc analyses compared to the primary analyses.

1. Differences in age vs. N100 amplitude

As difference in age between tinnitus and control subjects was found to be a significant moderator regarding the amplitude of the N100 component, a post hoc analysis was performed in order to characterize the relationship between age differences and N100 amplitude differences. Age difference was calculated as the average age of the control subjects substracted from the average age in the tinnitus group, so that positive age differences correspond to studies where control subjects were younger than tinnitus participants. A negative correlation (r = -0.10, *p* < 0.05) was identified between age difference and SMD for N100 amplitude, with more negative (i.e. larger) amplitudes corresponding to larger age differences (S1 Fig). After removing influential papers from this analysis (i.e. Jacobson et al., 1996 and Delb et al., 2008), this correlation was no longer significant. Notably, these two studies were characterized by the biggest age differences in this subgroup of papers, with control subjects being significantly younger than tinnitus patients.

[Insert S1 Fig about here]

**S1 Fig: Age difference is negatively correlated to the standardized mean difference for N100 amplitude (r = -0.10, *p* < 0.05).** Individual studies are represented by first author and year of publication. The grey dotted line represents the fitted regression line.

1. Publication bias

Egger’s regression tests were borderline significant for N100 amplitude (*p* = 0.06) and latency (*p* = 0.10), indicating a slight asymmetry of the funnel plots for these components (S2 Fig). Partially due to the relatively low sample size of these post hoc analyses per LAEP component, there is not enough statistical evidence to assume any publication bias, although the results of the Egger’s regression tests seem to suggest the presence of some publication bias for this component specifically.

[Insert S2 Fig about here]

**S2 Fig. Funnel plots for N100 amplitude (left) and latency (right) are borderline asymmetric.** Egger’s regression tests are borderline significant for N100 amplitude (*p* = 0.06) and latency (*p* = 0.10), indicating borderline asymmetry for these components. Asymmetric funnel plots are indicative of the presence of publication bias.

1. Results of sensitivity analyses

Several sensitivity analyses were performed to explore the influence of subtle differences in experimental conditions on the outcomes of the primary analyses. First, as only responses to target tones in oddball paradigms were included in the primary analysis, a secondary analysis was performed containing all available responses to non-target tones instead. This analysis resulted in a significant SMD for the amplitude of the N100 component (SMD = -0.55, *p* < 0.05), while consolidating the differences in P300 amplitude (SMD = -1.03, *p* < 0.001) and latency (SMD = 0.96, *p* < 0.01). No differences for any of the other LAEP components were found.

A second sensitivity analysis was performed to explore the possible influence of different active recording electrodes. As many authors reported results from the Fz electrode in addition to the Cz electrode, this analysis included amplitudes and latencies recorded at Fz instead of Cz, if available. Results from this analysis did not differ from the primary multivariate model. No significant differences were found except for P300 amplitude (SMD = -1.03, *p* < 0.001) and latency (SMD = 1.14, *p* < 0.001).

Some authors reported results from different subgroups of tinnitus patients that were compared to one control group. Because the primary analysis regarded these different subgroups as one tinnitus group with weighted averages and pooled standard deviation, a sensitivity analysis was performed including only one tinnitus subgroup for each of these papers (i.e. ‘high distress’, ‘tinnitus complainers’ and ‘bilateral tinnitus’). The inclusion of specific subgroups of tinnitus patients instead of a weighted average also did not alter the reported results (P300 amplitude: SMD = -0.93, *p* < 0.01; P300 latency: SMD = 0.88, *p* < 0.01; no other significant components).

An additional sensitivity analysis was performed to explore the influence of different experimental paradigms on the reported results. For this analysis, responses recorded in passive listening situations were included instead of those to active oddball scenarios, if available. Although the resulting SMD’s for P300 amplitude (SMD = -0.72, *p* < 0.05) and latency (SMD = 0.76, *p* < 0.01) were slightly smaller, they were still significant. No other component displayed a significant result between tinnitus patients and controls. Taken together, the results of these four different sensitivity analyses confirm the robustness of the outcomes of the primary multivariate model.

Finally, regarding the N100 and P200 components, some authors chose to report the amplitudes of these components separately, while others combined these amplitudes into one measure (sometimes called the ‘vertex potential’). The primary multivariate model described above included these components as they were reported in the original papers. However, alternative analyses were performed using either separate N100 and P200 amplitudes only, or combining these into one vertex potential amplitude. Results from these analyses did not differ from the primary multivariate model results. When including only N100 and P200 amplitude separately, results closely adhered to those of the primary model, with only P300 amplitude (SMD = 0.95, *p* < 0.01) and latency (SMD = 1.00, *p* < 0.01) differing significantly between tinnitus patients and controls. A second analysis including only combined vertex potentials also did not show a significant difference for this component, but only for the P300 amplitude (SMD = -1.03, *p* < 0.001) and latency (SMD = 1.15, *p* < 0.001).

To further investigate the possible effects of random factors, including age, gender and hearing level differences between tinnitus patients and controls, we performed a subgroup analysis on those studies where all of these factors were sufficiently reported. Only five out of the twenty-one total papers could be included in this subgroup analysis [1-5]. The P300 component was reported in none of these papers. Differences in CAEP components between tinnitus patients and controls were investigated, including the random factors of age, gender and hearing level as covariates. This analysis did not reveal any differences between tinnitus patients and controls for any of the included CAEP components (i.e. P50 amplitude and N100 and P200 amplitude and latency). None of the included covariates contributed significantly to the model.

1. Reduction of the dataset

In order to obtain a dataset containing one singular observation per LAEP component per paper, the following set of rules was followed:

- - If results recorded from multiple active electrodes were reported, results recorded at Cz were included for the final analysis.
  - If responses to both target and non-target tones were reported, only responses to target tones were included in the final model.
  - Results from multiple groups of tinnitus patients (bilateral vs. unilateral tinnitus, tinnitus complainers vs. non-complainers, high distress vs. low distress) were combined into a weighted average and a pooled standard deviation was calculated.
  - If results from multiple experimental paradigms were reported, only those results from oddball paradigms were included.

1. Mahmoudian S*, et al.* (2015) Alterations in auditory change detection associated with tinnitus residual inhibition induced by auditory electrical stimulation. *J Am Acad Audiol* 26(4):408-422.

2. Tugumia D, Samelli AG, Matas CG, Magliaro FC, & Rabelo CM (2016) Auditory training program in subjects with tinnitus. *CoDAS* 28(1):27-33.

3. Wise K, Kobayashi K, Magnusson J, Welch D, & Searchfield GD (2016) Randomized Controlled Trial of a Perceptual Training Game for Tinnitus Therapy. *Games for health journal* 5(2):141-149.

4. Jacquemin L*, et al.* (2019) An Exploratory Study on the Use of Event-Related Potentials as an Objective Measure of Auditory Processing and Therapy Effect in Patients With Tinnitus: A Transcranial Direct Current Stimulation Study. *Otol Neurotol* 40(9):e868-e875.

5. Mahmoudian S*, et al.* (2013) Central auditory processing during chronic tinnitus as indexed by topographical maps of the mismatch negativity obtained with the multi-feature paradigm. *Brain Res* 1527:161-173.

6. El-Minawi MS, Dabbous AO, Hamdy MM, & Sheta SM (2018) Does changes in mismatch negativity after tinnitus retraining therapy using tinnitus pitch as deviant stimulus, reflect subjective improvement in tinnitus handicap? *Hearing Balance Commun*.

7. Weisz N, Voss S, Berg P, & Elbert T (2004) Abnormal auditory mismatch response in tinnitus sufferers with high-frequency hearing loss is associated with subjective distress level. *BMC neuroscience* 5:8.

8. Kropp P*, et al.* (2012) Cortical habituation deficit in tinnitus sufferers: contingent negative variation as an indicator of duration of the disease. *Applied psychophysiology and biofeedback* 37(3):187-193.

9. Li Z*, et al.* (2016) Attentional Bias in Patients with Decompensated Tinnitus: Prima Facie Evidence from Event-Related Potentials. *Audiol Neurootol* 21(1):38-44.

10. Ku Y*, et al.* (2017) The gap-prepulse inhibition deficit of the cortical N1-P2 complex in patients with tinnitus: The effect of gap duration. *Hear Res* 348:120-128.

11. Campbell J, Bean C, & LaBrec A (2018) Normal hearing young adults with mild tinnitus: Reduced inhibition as measured through sensory gating. *Audiology research* 8(2):214.

12. Campbell J, LaBrec A, Bean C, Nielsen M, & So W (2019) Auditory Gating and Extended High-Frequency Thresholds in Normal-Hearing Adults With Minimal Tinnitus. *Am J Audiol* 28(1S):209-224.

13. Mahmoudian S*, et al.* (2013) Correlation between brain cortex metabolic and perfusion functions in subjective idiopathic tinnitus. *Int Tinnitus J* 18(1):20-28.

14. Shiraishi T*, et al.* (1991) Contingent negative variation enhancement in tinnitus patients. *American journal of otolaryngology* 12(5):267-271.

15. Attias J, Urbach D, Gold S, & Shemesh Z (1993) Auditory event related potentials in chronic tinnitus patients with noise induced hearing loss. *Hear Res* 71(1-2):106-113.

16. Attias J, Furman V, Shemesh Z, & Bresloff I (1996) Impaired brain processing in noise-induced tinnitus patients as measured by auditory and visual event-related potentials. *Ear Hear* 17(4):327-333.

17. Jacobson GP*, et al.* (1996) Electrophysiological indices of selective auditory attention in subjects with and without tinnitus. *Hear Res* 97(1-2):66-74.

18. Norena A, Cransac H, & Chery-Croze S (1999) Towards an objectification by classification of tinnitus. *Clin Neurophysiol* 110(4):666-675.

19. Jacobson GP & McCaslin DL (2003) A reexamination of the long latency N1 response in patients with tinnitus. *J Am Acad Audiol* 14(7):393-400.

20. Walpurger V, Hebing-Lennartz G, Denecke H, & Pietrowsky R (2003) Habituation deficit in auditory event-related potentials in tinnitus complainers. *Hear Res* 181(1-2):57-64.

21. Dornhoffer J, Danner C, Mennemeier M, Blake D, & Garcia-Rill E (2006) Arousal and attention deficits in patients with tinnitus. *Int Tinnitus J* 12(1):9-16.

22. Delb W*, et al.* (2008) Alterations in Event Related Potentials (ERP) associated with tinnitus distress and attention. *Applied psychophysiology and biofeedback* 33(4):211-221.

23. Santos Filha VA & Matas CG (2010) Late Auditory evoked potentials in individuals with tinnitus. *Braz J Otorhinolaryngol* 76(2):263-270.

24. Gabr TA, El-Hay MA, & Badawy A (2011) Electrophysiological and psychological studies in tinnitus. *Auris Nasus Larynx* 38(6):678-683.

25. Yang H*, et al.* (2013) The characteristic and changes of the event-related potentials (ERP) and brain topographic maps before and after treatment with rTMS in subjective tinnitus patients. *PLoS One* 8(8):e70831.

26. Houdayer E*, et al.* (2015) Involvement of cortico-subcortical circuits in normoacousic chronic tinnitus: A source localization EEG study. *Clin Neurophysiol* 126(12):2356-2365.

27. Hong SK, Park S, Ahn MH, & Min BK (2016) Top-down and bottom-up neurodynamic evidence in patients with tinnitus. *Hear Res* 342:86-100.

28. Gopal KV, Thomas BP, Nandy R, Mao D, & Lu H (2017) Potential Audiological and MRI Markers of Tinnitus. *J Am Acad Audiol* 28(8):742-757.

29. Mannarelli D*, et al.* (2017) Selective attentional impairment in chronic tinnitus: Evidence from an event-related potentials study. *Clin Neurophysiol* 128(3):411-417.

30. Asadpour A, Alavi A, Jahed M, & Mahmoudian S (2018) Cognitive Memory Comparison Between Tinnitus and Normal Cases Using Event-Related Potentials. *Frontiers in integrative neuroscience* 12:48.

31. Durai M, Sanders M, Kobayashi K, & Searchfield GD (2019) Auditory Streaming and Prediction in Tinnitus Sufferers. *Ear Hear* 40(2):345-357.

32. Morse K & Vander Werff KR (2019) Comparison of Silent Gap in Noise Cortical Auditory Evoked Potentials in Matched Tinnitus and No-Tinnitus Control Subjects. *Am J Audiol* 28(2):260-273.

33. Majhi SK, Khandelwal K, & Shrivastava MK (2019) Tinnitus and Cognition: Linked? *Indian J Otolaryngol Head Neck Surg* 71(Suppl 2):1426-1430.
