# Supplementary material for: Systematic review and meta-analysis of late auditory evoked potentials as a candidate biomarker in the assessment of tinnitus
Source: PLoS One. 2020 Dec 17;15(12):e0243785. doi: 10.1371/journal.pone.0243785 (PMC7746183; doi:10.1371/journal.pone.0243785)
Supplement: Attachment — Submitted filename: Response to the reviewers.docx [file pone.0243785.s005.docx]

**Comments from the academic editor**

**We would like to thank the academic editor for these additional comments. We have answered them in bold below.**

1. Please ensure that your manuscript meets PLOS ONE's style requirements, including those for file naming. The PLOS ONE style templates can be found at

https://journals.plos.org/plosone/s/file?id=wjVg/PLOSOne_formatting_sample_main_body.pdf and

https://journals.plos.org/plosone/s/file?id=ba62/PLOSOne_formatting_sample_title_authors_affiliations.pdf

**We have adjusted the manuscript and additional files according to the requirements.**

2.In your Data Availability statement, you have not specified where the minimal data set underlying the results described in your manuscript can be found. PLOS defines a study's minimal data set as the underlying data used to reach the conclusions drawn in the manuscript and any additional data required to replicate the reported study findings in their entirety. All PLOS journals require that the minimal data set be made fully available. For more information about our data policy, please see http://journals.plos.org/plosone/s/data-availability.

Upon re-submitting your revised manuscript, please upload your study’s minimal underlying data set as either Supporting Information files or to a stable, public repository and include the relevant URLs, DOIs, or accession numbers within your revised cover letter. For a list of acceptable repositories, please see http://journals.plos.org/plosone/s/data-availability#loc-recommended-repositories. Any potentially identifying patient information must be fully anonymized.

Important: If there are ethical or legal restrictions to sharing your data publicly, please explain these restrictions in detail. Please see our guidelines for more information on what we consider unacceptable restrictions to publicly sharing data: http://journals.plos.org/plosone/s/data-availability#loc-unacceptable-data-access-restrictions. Note that it is not acceptable for the authors to be the sole named individuals responsible for ensuring data access.

We will update your Data Availability statement to reflect the information you provide in your cover letter.

**We have uploaded the minimal data set underlying our results at the Zenodo repository (DOI: 10.5281/zenodo.4275525). We have saved the dataset and are waiting to publish it at the moment of publication of our manuscript.**

3.Thank you for stating the following financial disclosure:

 [This work was supported by an Applied Biomedical Research grant of the University of Antwerp (FWO T001618 N). The funders had no role in study design, data collection and analysis, decision to publish, or preparation of the manuscript.].

We note that one or more of the authors is affiliated with the funding organization, indicating the funder may have had some role in the design, data collection, analysis or preparation of your manuscript for publication; in other words, the funder played an indirect role through the participation of the co-authors. If the funding organization did not play a role in the study design, data collection and analysis, decision to publish, or preparation of the manuscript and only provided financial support in the form of authors' salaries and/or research materials, please do the following:

1. Review your statements relating to the author contributions, and ensure you have specifically and accurately indicated the role(s) that these authors had in your study. These amendments should be made in the online form.
2. Confirm in your cover letter that you agree with the following statement, and we will change the online submission form on your behalf:

“The funder provided support in the form of salaries for authors [insert relevant initials], but did not have any additional role in the study design, data collection and analysis, decision to publish, or preparation of the manuscript. The specific roles of these authors are articulated in the ‘author contributions’ section.”

**We agree with the statement made in option 2. This statement can be added as follows:**

**“The funder provided support in the form of salaries for authors EC, IJ, HV and AG, but did not have any additional role in the study design, data collection and analysis, decision to publish, or preparation of the manuscript. The specific roles of these authors are articulated in the ‘author contributions’ section.”**

**Comments from the reviewers**

**We would like to thank both reviewers for their thoughtful remarks and comments on our work. Below, we have addressed their comments point by point in bold. Additions or adjustments to the manuscript are added in** red**.**

Reviewer #1: This study aim to elucidate the potential role of the auditory evoked response as biomarker in the study of patients with tinnitus. Using a multivariate meta-analytic study, the authors analyze the differences in the Late auditory evoked potentials (LAEPs) between tinnitus patients and controls described in twenty-one articles. The main result was a significantly poorer response of the p300 component in tinnitus patients, manifested by a reduction in amplitude and an increase in latency.
The manuscript is novel, since there is no previously published meta-analysis for the same purpose. The writing is adequate and its reading is fluid to the reader. The authors had a correct control of the Bias and risks, which allows us to think about the development of studies aimed at establishing the p300 component as a biomarker in the study of tinnitus.

In my opinion, there are some minor issues that should be considered for the publication of the manuscript:

Abstract:
L29: I recommend to use the concept potential biomarker

**This has been altered in the manuscript.**

Discussion:

Since it does not appear in fig. 3, I recommend discussing how many of the articles analyzed showed significant differences in latency and / or amplitude of the p300, between the groups

**The following sentence was added to the manuscript at L236:**

Four and three out of the eight included individual papers reported significant differences regarding P300 amplitude and latency, respectively.

A variable not included in the study is the use of central nervous system depressant medications by tinnitus patients. I recommend discussing it as a potential bias of the study, since it is known that this class of drugs and others affect the auditory evoked response.

**It is certainly true that environmental factors such as the use of central nervous system medication can influence the auditory evoked response. However, since the majority of the included papers did not offer any information on drug use or pharmacological status of the participants, we could not include this factor in our risk of bias assessment. We have added the following section to the manuscript at L301 to clarify this issue:**

Moreover, careful attention should be payed to environmentally induced factors that are known to influence the auditory evoked response, such as the use of drugs that act on the central nervous system. As individuals in the tinnitus population might be more likely to use medication of this type, such as antidepressants, this could present an additional level of bias in the selected studies [1, 2]. The majority of the discussed authors did not provide any information on pharmacological status of the included participants. We strongly suggest a careful correction for this potentially confounding factor in future studies.

Figure:
I recommend improving the resolution of figures 1, 2 and 3 in the final manuscript

**New versions of the figures have been uploaded.**

Reference
Half of the articles summarized in Table 1 are not in the reference list (shiraishi 1991; Attias 1993, 1996; Jacobson 1996, 2003; Walpurger 2003; Dornhoffer 2006; Yang 2013; Houdayer 2015; Hong 2016, Gopal 2017)

**All references were added to the reference list.**

Reviewer #2: This is a systematic review of late auditory potential measurements in tinnitus patients. It is an important piece of work, and I am glad the authors took the effort to produce this review. The outcome is that in tinnitus patients, the P300 component of the auditory response is reduced in amplitudes and delayed in comparison to that in subjects without tinnitus. The authors dealt with the very heterogenous literature in a very thoughtful way.

A consequence is that studies have been dealt with in a heterogeneous way. Therefore
I invite the authors to present the final model of Fig. 3 in some sort of graphical way, clarifying the dependent (various P and N-peaks) and independent (tinnitus, hearing threshold, age) variables

**Thank you for this suggestion. We have added a graphical overview of the main results as Fig 5. We have added the following legend to the figure:**

**Fig 5. Graphical overview of the effects of increasing age, hearing loss and tinnitus on the LAEP waveform.** Illustration produced with BioRender.

Some additional comments:

L51: This paragraph is a bit odd, as it appears to describe how tinnitus patients are clinically assessed. The first sentence claims that this work is done by an ENT physician or an audiologist. However, in many tinnitus centers the patient is evaluated by both and additionally a psychologist. Here, you only need to briefly summarize the subjective measures (questionnaires etc). I think this paragraph is only intended to contrast the next paragraph, where objective measurements are introduced.

**We agree that some of the information in this paragraph seems superfluous. We have altered and shortened this paragraph as follows:**

Tinnitus patients are systematically evaluated at an outpatient clinic by an ear-nose-throat (ENT) physician, audiologist, and/or psychologist in a multidisciplinary setting. The impact of tinnitus on quality of life and the extent of accompanying symptoms are usually probed using questionnaires, such as […].

Fig. 2: Is this real data? I think it is just a cartoon to illustrate your computation. Please clarify in the text.

**The following sentence was added to the manuscript in the figure legend at L166:**

Illustration to clarify the used procedure.

L187: There is a funnel plot for P100 included in the supplemental material. As P300 is the most important outcome, I suggest to include funnel plots of P300 amplitude and delay. I suggest to put this in the main text, rather than in an appendix.

**Funnel plots for P300 amplitude and latency have been added as Figure 4. The following sentence has been added to the manuscript at L193:**

Representative funnel plots for P300 amplitude and latency are shown in Figure 4.

Fig. 3:
- It is confusing that the diamonds in the forest plot also appear as light grey diamonds overlapping with the results of individual studies. I suggest to remove those grey diamonds.

**The light grey diamonds have been removed from the figure.**

- Please double check the credibility intervals of P300 latency and amplitude. It seems odd that they are much larger than the 95% confidence intervals of the individual studies.

**Here, the credibility / prediction intervals are defined as the intervals where approximately 95% of the true outcomes are expected to fall. Therefore, if there is a large amount of variability in the observed outcomes (i.e. the estimates of the true outcomes), the credibility interval will be broad, as is the case for N100 and P300 latency and amplitude. However, if the observed outcomes are all in a similar range, the credibility interval is more narrow, as is the case for P50 amplitude. The following section has been added to the manuscript in the figure legend at L187:**

[…] error bars correspond to credibility/prediction intervals, defined as the intervals where approximately 95% of the true outcomes are expected to fall.

L207: The phrase 'Both of these authors' refers to two multi-author papers. Please correct.

**The manuscript has been altered as follows:**

The authors of both of these papers reported […].

L369: It is unclear to me whether age and hearing threshold were included as co-variates in the multivariate meta-analysis. This is important to do, and age and hearing loss is available for many studies. Please clarify. Please consider to perform a seperate analysis on the subclass of studies where age and hearing loss are both available.

**The final multivariate model did not include age and hearing levels as co-variates. These factors were initially added to the model, but were removed as they were found not to have a significant effect on the overall model outcome. However, it is important to recognize that these factors may have different effects on different CAEP components. This is why we chose to add these factors to the post hoc models investigating each component separately. For each of these models, random factors (differences in age, gender and hearing level between the groups) were added in a stepwise manner. We have added the following section to the manuscript at L410 to clarify this issue:**

Random factors (age, gender, hearing levels) were removed from the multivariate model, as they were found not to affect the overall model outcomes significantly.

**Only five papers included sufficient information on all relevant random factors: gender, age and hearing levels (pure tone averages) of participants in both groups. The P300 component was investigated in none of these five papers. Separate analysis of this subset of papers did not reveal any significant differences between tinnitus patients and controls in any of the included components (P50 amplitude, N100 amplitude and latency, and P200 amplitude and latency). Moreover, none of the included covariates (gender, age and hearing levels) contributed significantly to the model. For these reasons, we chose not to include this separate analysis in the main body of the text. We have added a paragraph to the Appendix at L100 to clarify this:**

To further investigate the possible effects of random factors, including age, gender and hearing level differences between tinnitus patients and controls, we performed a subgroup analysis on those studies where all of these factors were sufficiently reported. Only five out of the twenty-one total papers could be included in this subgroup analysis [3-7]. The P300 component was reported in none of these papers. Differences in CAEP components between tinnitus patients and controls were investigated, including the random factors of age, gender and hearing level as covariates. This analysis did not reveal any differences between tinnitus patients and controls for any of the included CAEP components (i.e. P50 amplitude and N100 and P200 amplitude and latency). None of the included covariates contributed significantly to the model.

1.	Deniz M, Bayazit YA, Celenk F, Karabulut H, Yilmaz A, Gunduz B, et al. Significance of serotonin transporter gene polymorphism in tinnitus. Otol Neurotol. 2010;31(1):19-24. doi: 10.1097/MAO.0b013e3181c2dcbc. PubMed PMID: 19924014.

2.	Gilles A, Van Camp G, Van de Heyning P, Fransen E. A Pilot Genome-Wide Association Study Identifies Potential Metabolic Pathways Involved in Tinnitus. Front Neurosci. 2017;11:71. doi: 10.3389/fnins.2017.00071. PubMed PMID: 28303087; PubMed Central PMCID: PMCPMC5332393.

3.	Norena A, Cransac H, Chery-Croze S. Towards an objectification by classification of tinnitus. Clin Neurophysiol. 1999;110(4):666-75. doi: 10.1016/s1388-2457(98)00034-0. PubMed PMID: 10378736.

4.	Gopal KV, Thomas BP, Nandy R, Mao D, Lu H. Potential Audiological and MRI Markers of Tinnitus. J Am Acad Audiol. 2017;28(8):742-57. doi: 10.3766/jaaa.16106. PubMed PMID: 28906245.

5.	Durai M, Sanders M, Kobayashi K, Searchfield GD. Auditory Streaming and Prediction in Tinnitus Sufferers. Ear Hear. 2019;40(2):345-57. doi: 10.1097/AUD.0000000000000620. PubMed PMID: 29933259.

6.	Morse K, Vander Werff KR. Comparison of Silent Gap in Noise Cortical Auditory Evoked Potentials in Matched Tinnitus and No-Tinnitus Control Subjects. Am J Audiol. 2019;28(2):260-73. doi: 10.1044/2018_AJA-18-0074. PubMed PMID: 31184965.

7.	Campbell J, LaBrec A, Bean C, Nielsen M, So W. Auditory Gating and Extended High-Frequency Thresholds in Normal-Hearing Adults With Minimal Tinnitus. Am J Audiol. 2019;28(1S):209-24. doi: 10.1044/2019_AJA-TTR17-18-0036. PubMed PMID: 31022362.
